# Supplementary material for: 3′dNTP Binding Is Modulated during Primer Synthesis and Translesion by Human PrimPol
Source: Int J Mol Sci. 2023 Dec 19;25(1):51. doi: 10.3390/ijms25010051 (PMC10778844; doi:10.3390/ijms25010051)
Supplement: Supplementary file 1 [file ijms-25-00051-s001.zip › ijms-2776200-supplementary.pdf]

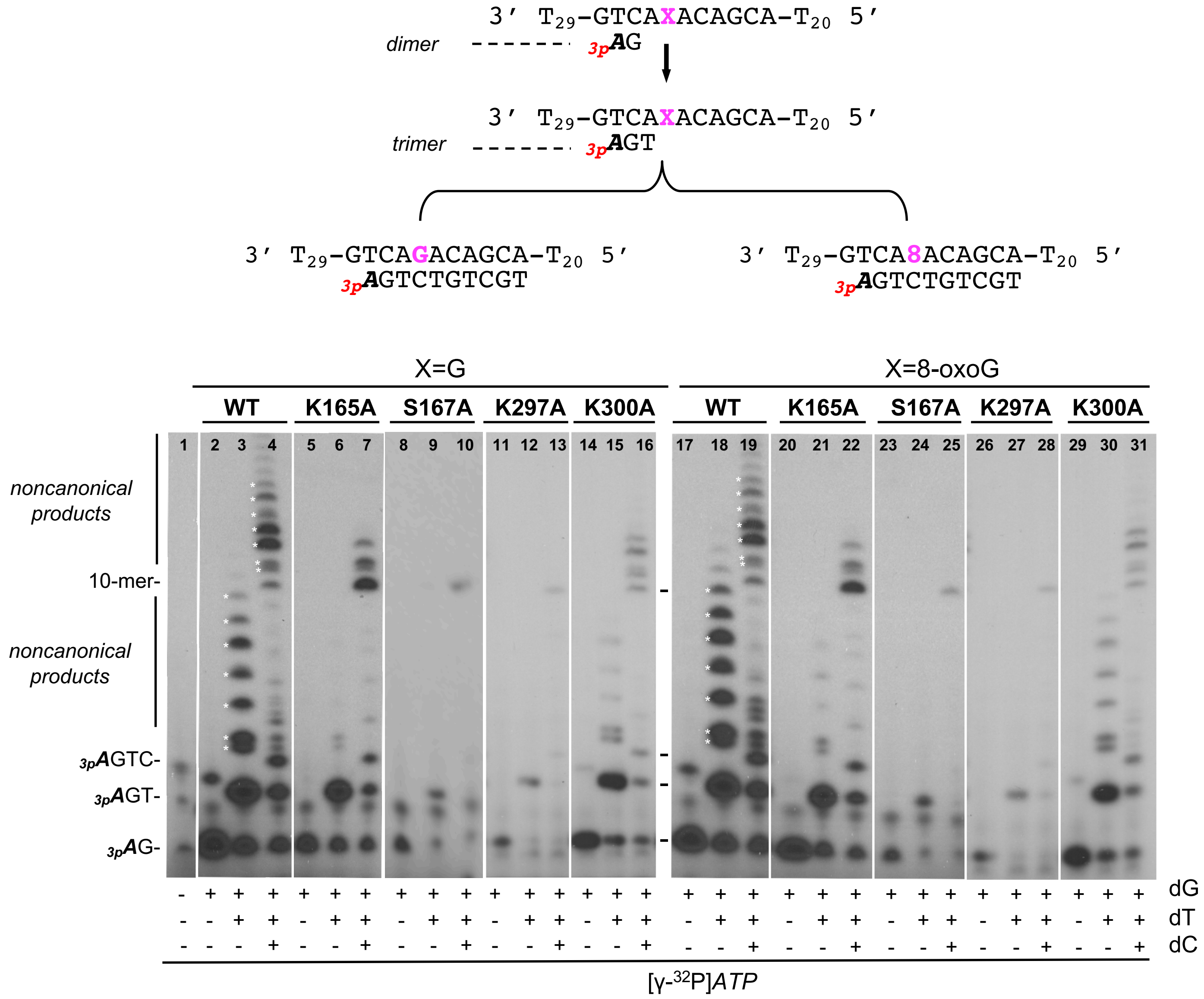

Figure S1. Single mutations affecting 3′dNTP binding do not destroy the ability of *Hs*PrimPol to tolerate an 8oxoG lesion. Primer formation and elonga tion by WT *Hs*PrimPol and mutants (400 nM) using 3′ T_{29}-GTCAXACAGCAT-T_{20} 5′ as a template, where X can be either a G (left panel) or an 8-oxoG lesion (right panel). *[γ-^{32}P]ATP* (16 nM) as the 5′ nucleotide, and dGTP, dTTP and dCTP (100 μM) as the 3′ incoming dNTPs were sequentially added, in the prese nce of 1 mM $MnCl_2$; non-canonical products are marked by asterisks.

As previously reported *Hs*PrimPol is well suited to tolerate 8-oxoG lesions (García-Gómez *et al.*, 2013 [3]; Martínez-Jiménez *et al.*, 2015 [34]), even during DNA primer synthesis (Díaz-Talavera, 2020 [33]). As shown here, *Hs*PrimPol used equally well both dG- and 8-oxoG-contaning templates (see the schematics) to start and elongate a DNA primer, giving rise to similar amounts of dimers, trimers, and fully elongated primers, and even non-canonical products. K300A mutant showed again a WT-like behavior when tolerating an 8-oxoG, producing similar canonical and non-canonical priming products (compare lanes 15-16 with 30-31). K165A, S167A, and K297A mutants could also tolerate 8-oxoG lesions during the priming reaction because longer products than 4-mer were observed, mirroring those on the control template (compare lines 7 *vs* 22, 10 *vs* 25, 13 *vs* 28). As expected, S167A and K297A mutants again synthesized a very limited and similar amount of product on both templates (compare lanes 10 & 25 (S167A), 13 & 28 (K297A) with lanes 4 & 19 (WT)).
